# The spontaneous resolution of a vortex vein varix: case report

**DOI:** 10.1186/s12886-021-01861-2

**Published:** 2021-02-24

**Authors:** Sara L Weidmayer, Hakan Demirci

**Affiliations:** 1 VA Ann Arbor Healthcare System, 2215 Fuller Road, 48105 Ann Arbor, Michigan USA; 2 WK Kellogg Eye Center, Department of Ophthalmology, University of Michigan, 1000 Wall Street, 40105 Ann Arbor, Michigan USA

**Keywords:** Vortex varices, Vortex varix, Vortex vein, Venous collateralization, Case report

## Abstract

**Background:**

The natural course of a vortex vein varix, though not well understood, has been known to remain stable. However, here we report a novel case of a vortex vein varix that resolved after an extended period of monitoring.

**Case presentation:**

An asymptomatic 96-year-old Caucasian man was found to have a vortex vein varix. At his previous examination 13 months prior, his fundus was normal. At 13 months of observation, his vortex vein varix become clinically undetectable. Further follow-up confirmed continued absence of the varix.

**Conclusion:**

This case demonstrates the development then clinical resolution of a vortex vein varix with no clear identifiable factors for its evolution. This case is novel and offers new insight into the natural history of some vortex vein varices, implicating venous congestion as an instigator and venous collateralization as its alleviator, suggesting that vortex vein varices are likely more common than previously reported since some may be temporary and under-identified.

## Background

The iris, ciliary body and choroidal venous systems drain into vortex veins before exiting the eye through scleral canals, then collect in the superior and inferior ophthalmic veins [1]. There are typically four or more vortex veins present, and physiologic vortex vein ampullae are present in about 44 % of patients [1]. Dilated vortex vein ampullae are known as vortex varices; they are rare and present generally as 1- to 3-disc diameter [2] brownish-red to gray colored elevations that cause no symptoms. These are benign, though they can cause mass effect on the overlying choriocapillaris, which may lead to changes in the retinal pigment epithelium (RPE) [3]. However, given their often dark and elevated presentation, variceal vortex vein ampullae may be easily mistaken for more concerning choroidal or subretinal problems, such as choroidal melanomas. Their development is not well understood, and they are not generally known to develop and later resolve. We report a novel case of a patient who developed a vortex vein varix that remained present for around 1 year, then resolved and remained clinically absent after extended additional follow up.

## Case presentation

**Fig. 1:**
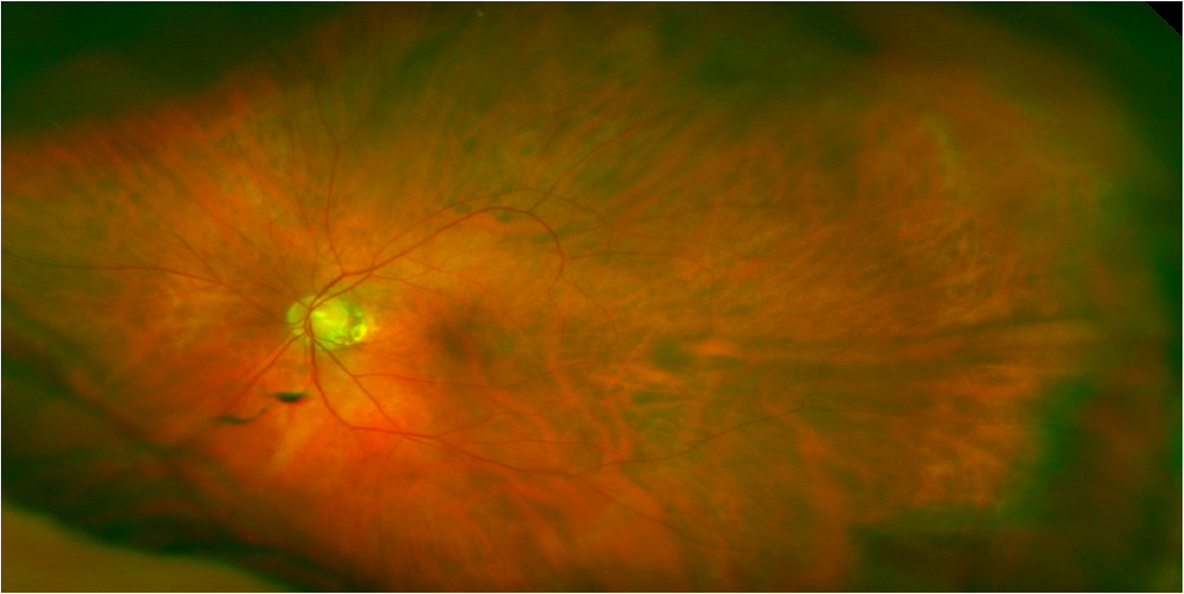
Unremarkable fundus photograph of the left eye, 13 months prior to the initial exam

**Fig. 2:**
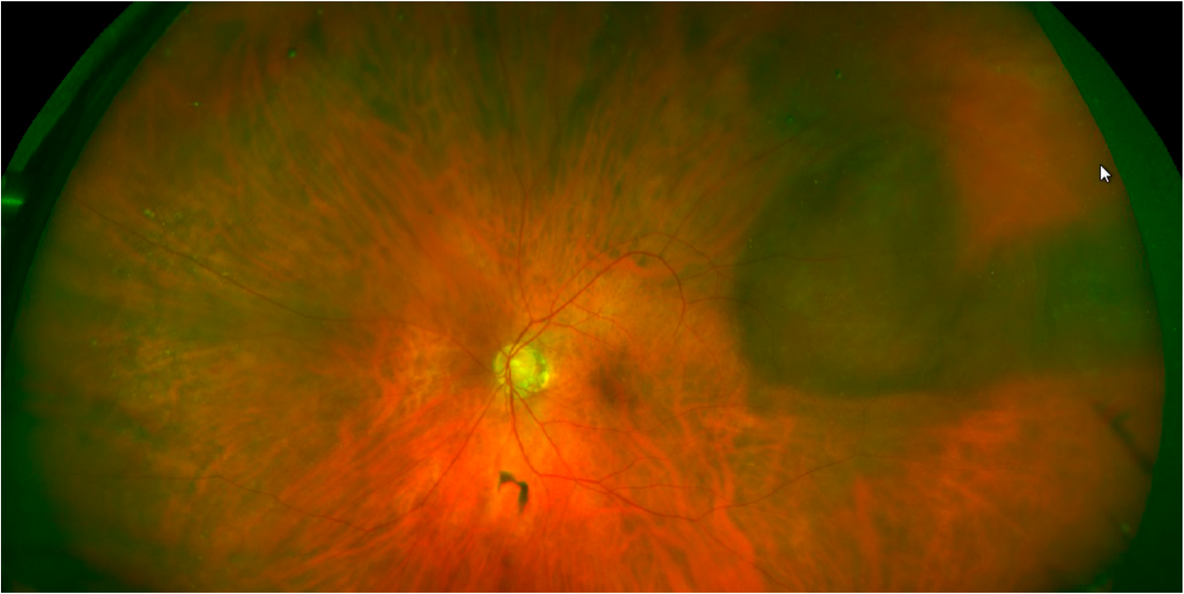
Fundus photograph of the left eye at initial presentation, demonstrating a 6 × 6 × 1.5 mm vortex vein varix in the superotemporal midperiphery

**Fig. 3:**
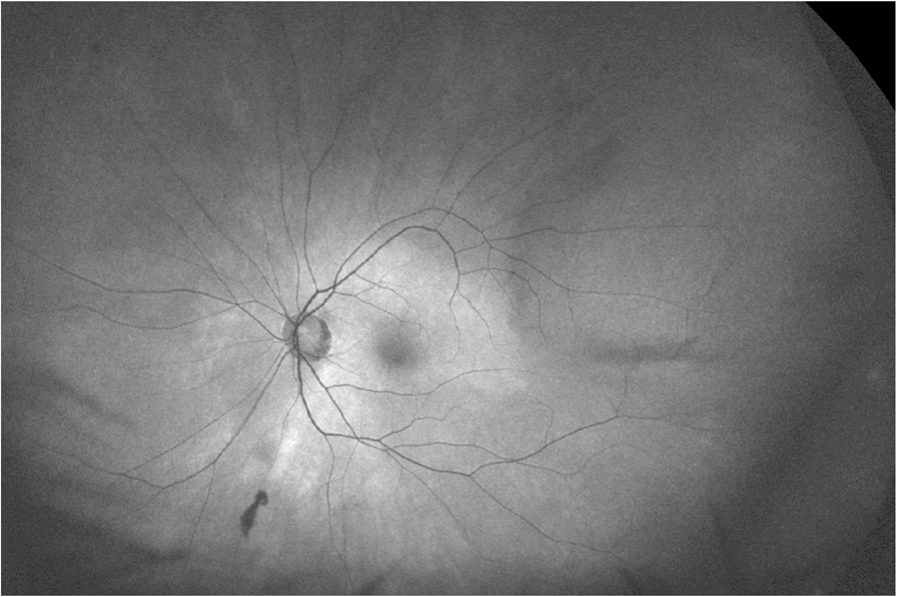
Autofluorescence imaging, where the lesion is isoautofluorescent with some shadowing along the elevated posterior and inferior margins

**Fig. 4:**
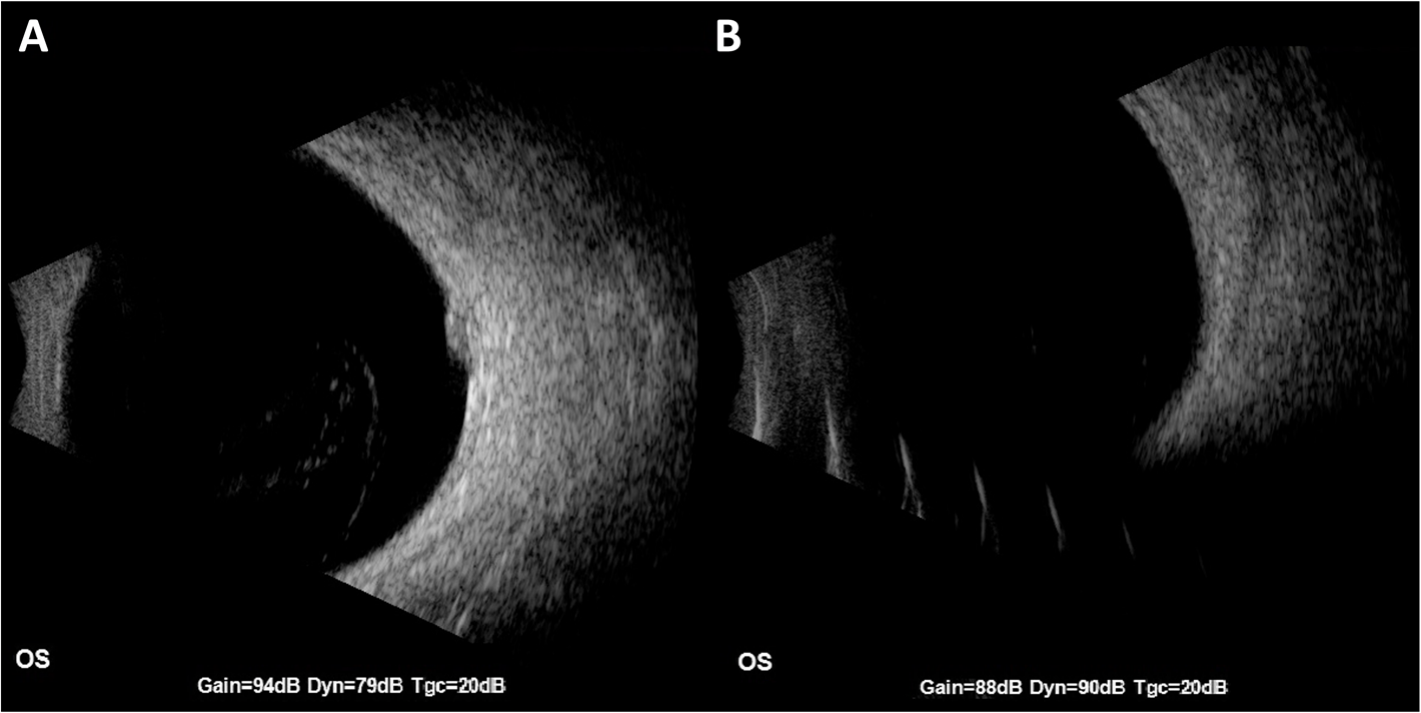
**a** B-scan ultrasonography showing a dome shaped, acoustically solid choroidal lesion with no extrascleral extension, and **b** with external pressure applied to the globe, showing the vortex varix flattened

**Fig. 5:**
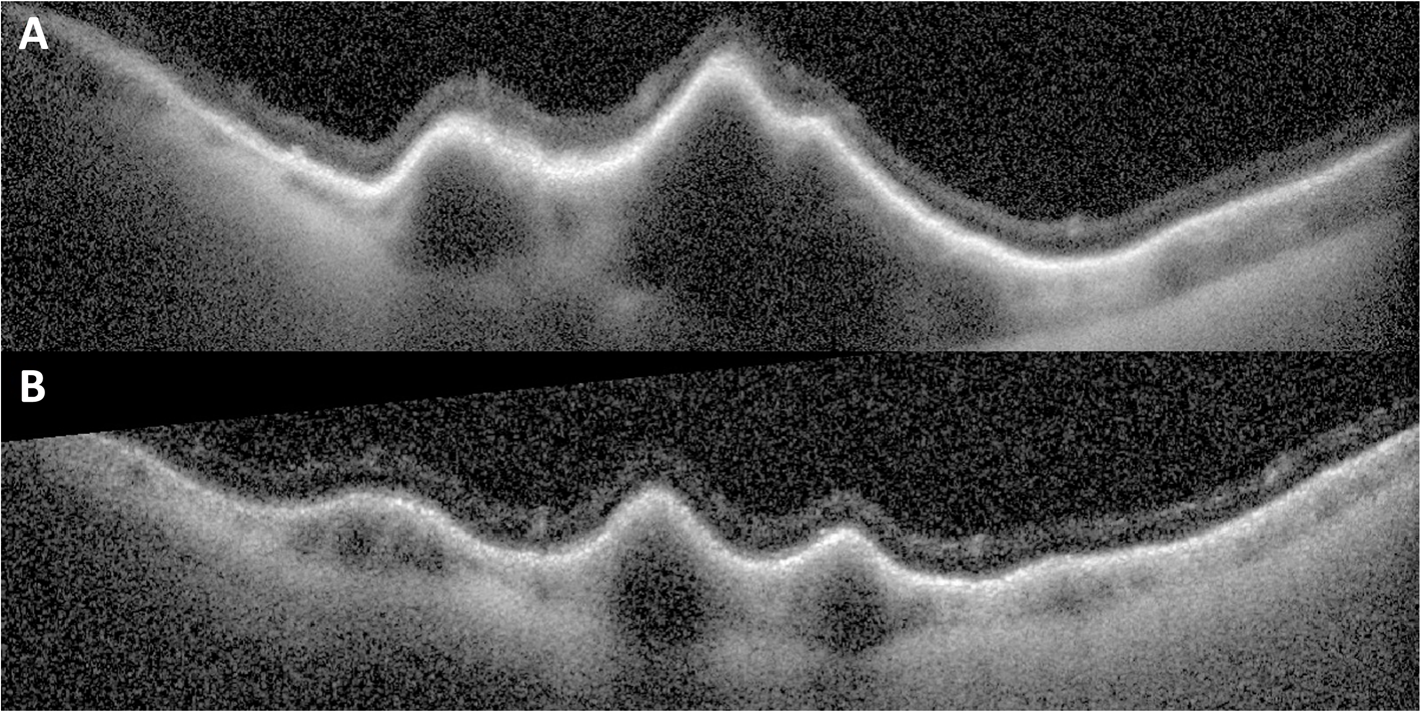
**a** Initial optical coherence tomography image through the vortex varix showing thickened choroidal vessels causing elevated- but otherwise undisturbed RPE and retina, and **b** relative shallowing of the vortex varix by OCT taken approximately 9 months after the initial exam

**Fig. 6:**
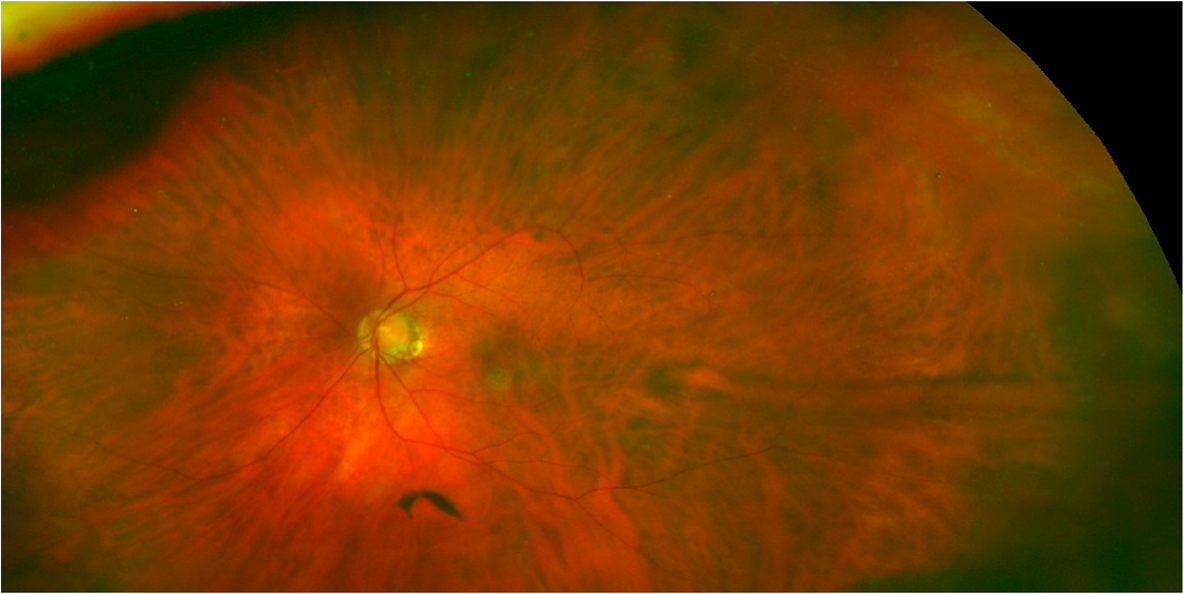
Fundus photograph of the left eye at a 3 month follow up interval after the vortex varix’s resolution, where the affected area remained flat

An asymptomatic 96-year-old white male with a history of hypertension, atrial fibrillation, basal cell- and squamous cell carcinoma of the scalp, and vitamin B12 deficiency presented for a routine eye exam. His last eye exam had been 13 months prior and was unremarkable (Fig. 1). His best-corrected visual acuity was 20/20 right eye (OD), 20/20 − 2 left eye (OS). Fundus examination of the left eye revealed a darkly colored choroidal elevation without subretinal fluid, drusen or orange pigment in the superotemporal midperiphery, measuring 6 × 6 × 1.5 mm (Fig. 2). The lesion was isoautofluorescent relative to adjacent structures (Fig. 3). B-scan showed a dome-shaped, elevated acoustically-solid choroidal lesion with no extrascleral extension (Fig. 4a). It flattened with increased external pressure applied to the globe (Fig. 4b), consistent with benign a vortex vein varix and excluding other differentials, such as melanoma or other solid lesions. Optical coherence tomography (OCT) scans were consistent with a vortex vein varix superotemporally, indicating thickened choroidal vessels causing elevated RPE contour with normal overlying retina (Fig. 5A). During a 9-month period of observation, this lesion remained largely stable clinically and showed comparative shallowing by OCT (Fig. 5b). At a visit thirteen months from the initial evaluation, the elevated vortex varix had entirely flattened, even when the patient looked superotemporally. There was no recurrence during an additional 3-month interval (Fig. 6).

## Discussion and conclusions

As was the case with this patient, vortex varices may initially appear very alarming, simulating morbid lesions. Given that he was anticoagulated, a localized choroidal hematoma, which are thought to form from a ruptured short- or long ciliary artery branch, is a reasonable differential since they also appear as homogenous dark, dome-shaped choroidal lesions. This patient had no recent history of ocular surgery or trauma that might incite a choroidal hematoma, which usually form post-operatively or post-traumatically, though his age, low intraocular pressure (IOP) and anticoagulated state would increase the risk of spontaneous development. However, unlike in this patient, localized choroidal hematomas often present with pain or vision changes, often show prominent overlying choroidal folds, are hypoechoic on B-scan ultrasonography and usually resolve in 1 to 2 months [4].

Dynamic clinical evaluation and ancillary imaging are helpful tools in the diagnosis of vortex varices. Vortex varices collapse when digital pressure is applied to the globe: this external pressure increases the IOP exceeding the normal pressure gradient compared to the choroidal venous pressure, [5] resulting in flattening of the varix. This phenomenon is clinically visible during dynamic fundoscopy [3] and has also been demonstrated with dynamic enhanced depth imaging OCT [6] and B-scan ultrasonography [7]. Similarly, vortex varices may distend and engorge in head-down-[5] or prone positioning due to venous stasis caused by increased orbital pressure, [8] with Valsalva maneuvers due to increased peripheral venous pressure [5] or when the patient’s gaze is directed towards the varix, likely related to extrascleral venous crimping [1]. Venous reflux into the vortex varix may also be seen with jugular vein compression [5]. These are all useful clinical tools to assist in discriminating these vortex varices from other differential diagnoses; however, indocyanine green angiography is generally confirmatory, showing early maximum fluorescence with homogenous filling of the vortex ampulla, and total late washout [9].

In this patient, there are no clear factors that incited the development- or later resolution of his large vortex varix. He had few insignificant medication changes surrounding this time, and none appeared to impact this variceal event. While arterial blood pressure can quickly change, sometimes as a result of medication changes, venous pressure is far less volatile. He had no known medical conditions or events causing- or that could cause notable changes in venous pressure, such as gross changes in blood volume or cardiac output, that could contribute to vortex varix formation or resolution.

This patient’s IOP was generally low. In the 4 years prior to the vortex varix development, his average IOP in our clinic had been 9 mmHg OD and 7.75 mmHg OS (n = 4). During all follow ups in this series, it remained in this range. There are currently no reports of vortex varix development related to low or hypotonous IOP. Given this patient’s low IOP, increases in venous pressure could, in theory, contribute to vortex varix formation. Yet interestingly, at the exam when his IOP was its lowest recorded at only 6 mmHg OD and 7 mmHg OS, the vortex varix remained flat.

Most cases of vortex vein varices have been reported as incidental findings, or having been mistaken for more concerning choroidal lesions, but have not been noted to change over time. However, there has been one report of a macular vortex varix in a highly myopic young male patient, which continued to enlarge along with increasing axial length with age [10]. Also, induction- then resolution of a vortex varix has twice been reported in association with posterior nodular scleritis, [9, 11] suggesting that compression (due to inflammation in the case of scleritis) and congestion of adjacent scleral drainage may lead to variceal formation [9, 11]. However, to our knowledge, this case of sustained vortex varix development then self-resolution in the absence of any clear inciting factor is novel. In all, this elevated vortex varix was photographically documented to be present during a 9-month span and was resolved by the next follow up 4 months later. It remained absent for another 3-month interval thereafter.

Now knowing that vortex vein varices may develop then resolve with observation serves as a basis for pathophysiologic inquiry. It is theorized that a near-by downstream thromboembolus in this vortex vein’s collection route may have led to congestion of venous blood and this vortex varix formation. Choroidal veins are known to have significant plasticity, allowing them to develop alternate drainage routes via venovenous anastamoses to compensate for occluded vortex veins by connecting to nearby intact vortex veins [12]. Collateralization is also true for- and often seen in retinal venous occlusive disease. It is proposed here that the development of choroidal venovenous anastomoses or perhaps even the development extrascleral venous collaterals occurred after a thromboembolus caused bottlenecking, which alleviated vortex varix congestion and thus lead to resolution of the varix. This patient was anticoagulated with warfarin with consistent international normalized ratios (INR), so thromboembolus formation was improbable, but is a plausible explanation for sustained vortex varix formation then resolution in this elderly man. Since, until now, vortex vein varix resolution was not a foreseeable possibility, this was not confirmed via angiography, and is a limitation of this case report. However, to evaluate this on future cases, serial wide-field indocyanine green (ICG) angiography of the choroid- or serial angiography of the episcleral vessels external to the region of a vortex vein varix could be performed.

In summary, we present a 96-year-old male who presented with a varicose vortex vein varix that had not been present 13 months prior and resolved in another 13-month period, with no recurrence 3 months thereafter. There were no notable changes in his ocular or medical history during this time period. This novel case demonstrates that vortex vein varices may develop and later resolve, pointing to venous congestion as a cause and venous collateralization as its alleviant, and given this possible transiency that vortex vein varices are likely under-identified and more common than historically thought.

## Data Availability

Not Applicable.

## Abbreviations

ICG: Indocyanine green
INR: International normalized ratio
IOP: Intraocular pressure
OCT: Optical coherence tomography
OD: Right eye
OS: Left eye
RPE: Retinal pigment epithelium
